# Clofazimine Modulates the Expression of Lipid Metabolism Proteins in *Mycobacterium leprae-*Infected Macrophages

**DOI:** 10.1371/journal.pntd.0001936

**Published:** 2012-12-06

**Authors:** Yang Degang, Takeshi Akama, Takeshi Hara, Kazunari Tanigawa, Yuko Ishido, Masaichi Gidoh, Masahiko Makino, Norihisa Ishii, Koichi Suzuki

**Affiliations:** 1 Leprosy Research Center, National Institute of Infectious Diseases, Higashimurayama, Tokyo, Japan; 2 Department of Leprosy and Infectious Diseases, Shanghai Skin Disease Hospital, Shanghai, China; University of California San Diego School of Medicine, United States of America

## Abstract

*Mycobacterium leprae (M. leprae)* lives and replicates within macrophages in a foamy, lipid-laden phagosome. The lipids provide essential nutrition for the mycobacteria, and *M. leprae* infection modulates expression of important host proteins related to lipid metabolism. Thus, *M. leprae* infection increases the expression of adipophilin/adipose differentiation-related protein (ADRP) and decreases hormone-sensitive lipase (HSL), facilitating the accumulation and maintenance of lipid-rich environments suitable for the intracellular survival of *M. leprae*. HSL levels are not detectable in skin smear specimens taken from leprosy patients, but re-appear shortly after multidrug therapy (MDT). This study examined the effect of MDT components on host lipid metabolism *in vitro*, and the outcome of rifampicin, dapsone and clofazimine treatment on ADRP and HSL expression in THP-1 cells. Clofazimine attenuated the mRNA and protein levels of ADRP in *M. leprae*-infected cells, while those of HSL were increased. Rifampicin and dapsone did not show any significant effects on ADRP and HSL expression levels. A transient increase of interferon (IFN)-β and IFN-γ mRNA was also observed in cells infected with *M. leprae* and treated with clofazimine. Lipid droplets accumulated by *M. leprae*-infection were significantly decreased 48 h after clofazimine treatment. Such effects were not evident in cells without *M. leprae* infection. In clinical samples, ADRP expression was decreased and HSL expression was increased after treatment. These results suggest that clofazimine modulates lipid metabolism in *M. leprae*-infected macrophages by modulating the expression of ADRP and HSL. It also induces IFN production in *M. leprae*-infected cells. The resultant decrease in lipid accumulation, increase in lipolysis, and activation of innate immunity may be some of the key actions of clofazimine.

## Author Summary

Leprosy, caused by *Mycobacterium leprae (M. leprae)*, is an ancient infectious disease that remains the leading infectious cause of disability. After infection, *M. leprae* lives inside host macrophages that contain a large amount of lipids, which is thought to be an essential microenvironment for *M. leprae* to survive in host cells. *M. leprae* infection increases lipid accumulation in macrophages and decreases the metabolic breakdown of lipids (catabolism). In addition, the treatment of leprosy with multidrug therapy (MDT) reverses the effect of infection on the modulation of lipid metabolism. We therefore aimed to use cultured human macrophage cells to determine which of the three MDT drugs (clofazimine, dapsone, or rifampicin) is responsible for this effect. We found that only clofazimine affects lipid accumulation and catabolism in *M. leprae*-infected cells *in vitro*. The amounts of lipids accumulated in the cells decreased when clofazimine was added to the cell culture medium. Clofazimine also activated immune responses in *M. leprae*-infected cells. These results suggest that the effectiveness of clofazimine against leprosy is due to the modulation of lipid metabolism and activation of immune reactions in *M. leprae*-infected host cells.

## Introduction

Leprosy is a chronic infectious disease caused by *Mycobacterium leprae* (*M. leprae*), which is a typical intracellular pathogen that parasitizes tissue macrophages (histiocytes) and Schwann cells of the peripheral nerves of the dermis. Although its prevalence has declined over the last several decades due to the introduction of multi-drug therapy (MDT) by the World Health Organization (WHO), leprosy remains a major public health problem in many developing countries: In 2010, 228,474 new cases were registered worldwide [1]. Based on their clinical, histological and immunological manifestations, leprosy patients are classified into five groups that comprise one continuous spectrum: Tuberculoid (TT), Borderline Tuberculoid (BT), Borderline (BB), Borderline Lepromatous (BL) and Lepromatous (LL) [2]. LL is characterized by widespread skin lesions containing numerous bacilli that live in the foamy or enlarged lipid-filled phagosome within macrophages. Schwann cells in LL nerves also have the foamy, lipid-laden appearance that favors mycobacterial survival and persistence. In Schwann cells, *M. leprae* infection-induced biogenesis of lipid droplets correlates with increased prostaglandin E2 (PGE2) and interleukin-10 (IL-10) secretion, which is essential for leprosy pathogenesis [3], [4]. Although lipid-laden macrophages are also observed in other mycobacterial infections, including tuberculosis [5], [6], the amount of lipid and the number of infected macrophages are most prominent in cases of LL [7], [8].

The PAT protein family is named after three of its members: perilipin, adipophilin/adipose differentiation-related protein (ADRP), and tail-interacting protein of 47 kDa (TIP47). PAT family members are responsible for the transportation of lipids and the formation of lipid droplets in a variety of tissues and cultured cell lines, including adipocytes [9]–[12]. ADRP selectively increases the uptake of long chain fatty acids and has an essential role in fatty acid transport [13], [14]. Hormone-sensitive lipase (HSL), as the first enzyme identified in the induction of lipo-catabolic action initiated by hormones, is the predominant lipase effector of catecholamine-stimulated lipolysis in adipocytes [15]. Therefore, ADRP and HSL have opposing functions, i.e., lipid accumulation vs. its degradation. ADRP and HSL also play important roles in lipid accumulation in *M. leprae*-infected macrophages [8], [16]. *M. leprae* infection increased the expression of ADRP mRNA and protein, facilitating the accumulation and maintenance of a lipid-rich environment suitable for intracellular survival [8]. Conversely, HSL expression was suppressed in macrophages infected with *M. leprae* [16]. These results suggest that both ADRP and HSL influence the lipid-rich environment that favors *M. leprae* parasitization and survival in infected host cells. In our previous study, HSL expression was not detectable in slit-skin smear specimens from non-treated LL and BL patients, but it re-appeared shortly after MDT treatment [16]. However, how treatment modulates HSL expression is not clear. In the present study, we determine the effect of MDT components on host lipid metabolism by investigating the influence of rifampicin, dapsone and clofazimine on the expression of ADRP and HSL in THP-1 cells.

## Materials and Methods

### Ethic statement

Human specimens were used according to the guidelines approved by the Ethical Committee of the National Institute of Infectious Diseases (Tokyo, Japan). All samples were anonymized before use.

### Drugs

Clofazimine (Sigma-Aldrich Co., St. Louis, MO), rifampicin (Wako Pure Chemical Industries Ltd., Osaka, Japan) and dapsone (Wako Pure Chemical Industries Ltd.) were dissolved in dimethyl sulfoxide (DMSO) and stored at 4°C. The final concentration used in the culture medium was 8.0 µg/ml rifampicin, 5.0 µg/ml dapsone or 2.0 µg/ml clofazimine.

### *M. leprae* isolation and cell culture

Hypertensive nude rats (SHR/NCrj-rnu), infected with the Thai53 strain of *M. leprae* [17], [18] were kindly provided by Dr. Y. Yogi of the Leprosy Research Center, National Institute of Infectious Diseases. Japan. The protocol was approved by the Experimental Animal Committee, of the National Institute of Infectious Diseases, Tokyo, Japan (Permit Number: 206055). Animal studies were carried out in strict accordance with the recommendations from Japan's Animal Protection Law. *M. leprae* was isolated as previously described [19], [20]. The human premonocytic cell line THP-1 was obtained from the American Type Culture Collection (ATCC; Manassas, VA). The cells were cultured in six-well plates in RPMI medium supplemented with 10% charcoal-treated fetal bovine serum (FBS), 2% non-essential amino acids, 100 IU/ml penicillin and 100 µg/ml streptomycin at 37°C in 5% CO_2_ [7], [8]. Typically, 3×10^7^ bacilli were added to 3×10^6^ THP-1 cells (multiplicity of infection: MOI = 10).

### RNA preparation and reverse transcription polymerase chain reaction (RT-PCR)

Total RNA from cultured cells was prepared using RNeasy Mini Kits (Qiagen Inc., Valencia, CA) as described previously [7], [8]. Total RNA preparation from slit-skin smear samples was performed as described [8], [16]. Briefly, stainless steel blades (Feather Safety Razor Co., Osaka, Japan) used to obtain slit-skin smear specimens were rinsed in 1 ml of sterile 70% ethanol. The tube was then centrifuged at 20,000×g for 1 min at 4°C. After removing the supernatant, RNA was purified with the same protocol that was used for cultured cells. The RNA was eluted in 20 µl of elution buffer and treated with 0.1 U/µl DNase I (TaKaRa Bio, Kyoto, Japan) at 37°C for 60 min to degrade any contaminating genomic DNA. All RNA samples had an OD260/280 of 1.8–2.0 and an OD260/230 >1.8. RNA sample quality was also confirmed using denaturing agarose gel electrophoresis and the Agilent 2100 Bioanalyzer (Agilent Technologies, Santa Clara, CA) (Fig. S1). Total RNA from each sample was reverse-transcribed to cDNA using a High Capacity cDNA Reverse Transcription Kit (Applied Biosystems, Foster City, CA) with random primers [8], [16]. The following primers were used to amplify specific cDNAs: ADRP: 5′-TGTGGAGAAGACCAAGTCTGTG-3′ (forward) and 5′-GCTTCTGAACCAGATCAAATCC-3′ (reverse); HSL: 5′-CTCCTCATGGCTCAACTCCTTCC-3′ (forward) and 5′-AGGGGTTCTTGACTATGGGTG-3′ (reverse); interferon (IFN)-β: 5′-TGCTCTCCTGTTGTGCTTCTCCAC-3′ (forward) and 5′-CAATAGTCTCATTCCAGCCAGTGC-3′ (reverse); IFN-γ: 5′-GCAGAGCCAAATTGTCTCCTTTTAC-3′ (forward) and 5′-ATGCTCTTCGACCTCGAAACAGC-3′ (reverse) and actin: 5′-AGCCATGTACGTAGCCATCC-3′ (forward) and 5′-TGTGGTGGTGAAGCTGTAGC-3′ (reverse). Touchdown PCR was performed using a PCR Thermal Cycler DICE (TaKaRa Bio, Tokyo, Japan) [7], [8]. Briefly, the PCR mixture was first denatured for 5 min at 94°C, followed by 20 cycles of three-temperature PCR consisting of a 30-sec denaturation at 94°C, a 30-sec annealing that started at 65°C and decreased 0.5°C every cycle to 55°C, and a 45-sec extension at 72°C. An additional 10 cycles were performed for ADRP and β-actin, and 14 cycles for HSL with a fixed annealing temperature of 55°C. The products were analyzed by 2% agarose gel electrophoresis.

### Protein preparation and Western blot analysis

Cellular protein was extracted and analyzed as previously described [16], [21]. Briefly, cells were lysed in a lysis buffer containing 50 mM HEPES, 150 mM NaCl, 5 mM EDTA, 0.1% NP40, 20% glycerol, and protease inhibitor cocktail (Complete Mini, Roche, Indianapolis, IN) for 1 h. After centrifugation, the supernatant was transferred and 10 µg of protein was used for analysis. Cellular proteins were mixed with 4× LDS sample buffer and 10× reducing agent (Invitrogen, Life Technologies, Carlsbad, CA) and incubated for 10 min at 70°C prior to electrophoresis. Proteins were separated on NuPage 4–12% Bis Tris Gels and transferred using an iBlot Gel Transfer Device (Invitrogen). The membrane was washed with PBST (phosphate buffered saline (PBS) with 0.1% Tween 20), blocked in blocking buffer (PBST containing 5% skim milk) overnight, and then incubated with either rabbit anti-ADRP antibody (Santa Cruz Biotechnology Inc., Santa Cruz, CA; 1∶2,000 dilution), rabbit anti-HSL antibody (Cell Signaling Technology, Danvers, MA; 1∶1,000 dilution) or goat anti-β-actin antibody (Santa Cruz; dilution 1∶2,000). After washing with PBST, the membrane was incubated for 1 h with biotinylated donkey anti-rabbit antibody for ADRP and HSL (GE Healthcare, Fairfield, CT; 1∶2,000 dilution) or biotinylated donkey anti-goat antibody for β-actin (Millipore, Billerica, MA; dilution 1∶10,000) followed by streptavidin-HRP (GE Healthcare; 1∶10,000 dilution) for 1 h. The signal was developed using ECL Plus Reagent (GE Healthcare).

### Lipid staining

THP-1 cells were grown on glass coverslips in 24-well plates for 24 h, before the culture medium was exchanged with RPMI 1640 containing *M. leprae* and clofazimine. Control and drug-treated THP-1 cells were fixed in 10% formalin for 10 min. They were then washed with Dulbecco's PBS (DPBS) and balanced with 60% isopropanol for 1 min before staining with oil-red-O (Muto Pure Chemicals, Tokyo, Japan) for 10 min. The cells were counterstained with hematoxylin for 5 min followed by ethanol dehydration and coverslip sealing.

### Immunohistochemistry

Archived formalin-fixed, paraffin-embedded tissue sections were subjected to immunohistochemical staining as described [7]. Briefly, deparaffinized sections were heated in 1 mM NaOH at 120°C for 5 min for antigen retrieval. They were then washed with PBST and blocked in blocking buffer (DAKO, Carpinteria, CA) for 10 min, and then incubated with either anti-ADRP antibody (Santa Cruz Biotechnology Inc.; 1∶200 dilution) or anti-HSL antibody (Cell Signaling Technology; 1∶100 dilution), for 1 h at room temperature. After washing the slides with PBST, peroxidase-labeled streptavidin-biotin method was employed using the LSAB2 kit (DAKO) and 3,3-diaminobenzidine tetrahydrochloride (DAB) for the staining of ADRP. Tyramide signal amplification (TSA)-HRP method was utilized to amplify HSL staining signals using the TSA Biotin System (PerkinElmer, Inc., Waltham, MA) according to the manufacturer's protocol. Sections were then stained using carbol fuchsin to visualize acid-fast mycobacteria and counterstained with hematoxylin.

### Others

All experiments were repeated at least three times. Since the replicates produced essentially the same outcomes, representative results from these independent experiments are shown in the figures.

## Results

### Clofazimine decreases ADRP and increases HSL mRNA levels in macrophages infected with *M. leprae*

**Figure 1:**
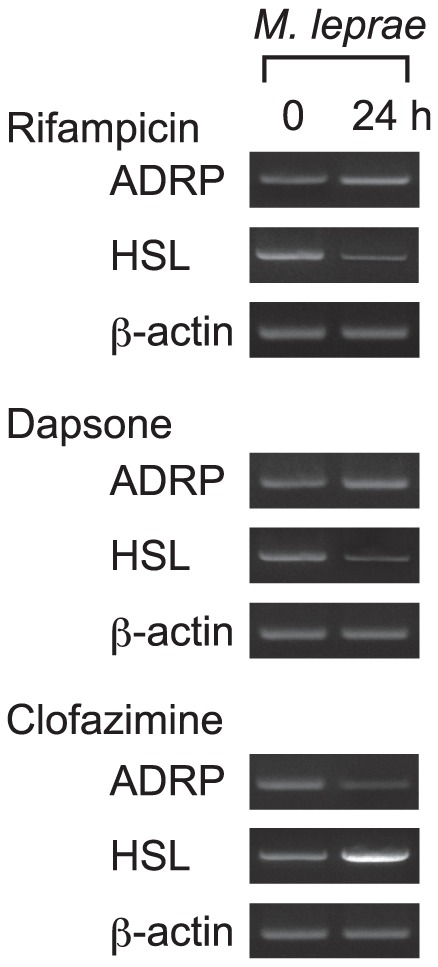
Expression of ADRP and HSL is modulated by clofazimine in THP-1 cells infected with *M. leprae*. THP-1 cells were cultured in six-well plates with culture medium containing either 8.0 µg/ml rifampicin, 5.0 µg/ml dapsone or 2.0 µg/ml clofazimine with *M. leprae* infection (MOI = 10). After incubating for 24 h, total RNA was purified and RT-PCR analysis of ADRP, HSL and β-actin was performed. Representative results from three independent experiments are shown.

The effect of MDT drugs on lipid metabolism in *M. leprae*-infected macrophages was examined by infecting human premonocytic THP-1 cells with *M. leprae* (MOI = 10) in the presence of 8.0 µg/ml rifampicin, 5.0 µg/ml dapsone or 2.0 µg/ml clofazimine for 24 h. Total RNA was isolated and RT-PCR analysis was performed to evaluate possible changes in ADRP and HSL mRNA levels. In our previous studies, *M. leprae* infection has been shown to increase ADRP and decrease HSL expression, which will in turn increase the lipid accumulation that is thought to contribute to maintaining a phagosome environment which permits *M. leprae* to parasitize tissue macrophages [8], [16]. However, when *M. leprae*-infected THP-1 cells were treated with clofazimine, ADRP expression levels decreased and HSL expression increased (Fig. 1). Rifampicin and dapsone did not show significant effects on the mRNA expression of ADRP, while they decreased HSL expression by augmenting the effect of *M. leprae* infection.

### The effect of clofazimine is specific only for *M. leprae*-infected cells

**Figure 2:**
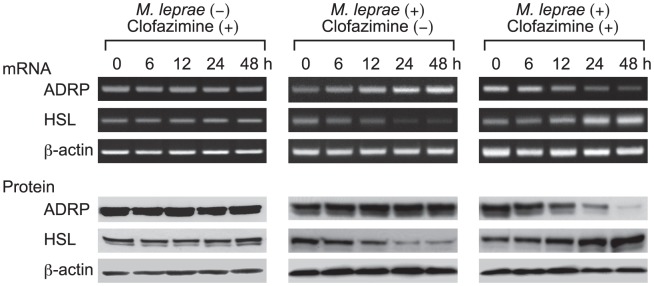
Only *M. leprae*-infected THP-1 cells are susceptible to clofazimine. THP-1 cells were cultured in six-well plates with or without 2.0 µg/ml clofazimine in the presence or absence of *M. leprae* infection (MOI = 10). After incubating for the indicated period of time, total RNA and total cellular protein were purified and RT-PCR and Western blot analyses of ADRP, HSL and β-actin were performed. Representative results from three independent experiments are shown.

To further evaluate the effect of clofazimine on ADRP and HSL expression, THP-1 cells were treated with clofazimine in the presence or absence of *M. leprae* infection for 6, 12, 24 and 48 h. Total RNA and cellular protein were extracted and used for RT-PCR analysis and Western blot analysis, respectively. Linearity of the RT-PCR amplifications of ADRP, HSL and β-actin was confirmed by serial dilution of RNA samples and densitometric analysis of the bands (Fig. S2). RT-PCR showed that clofazimine alone had no effect on ADRP and HSL mRNA levels in control THP-1 cells (Fig. 2, left panel). Consistent with previous reports, ADRP mRNA expression was increased and HSL mRNA expression was decreased when THP-1 cells were infected with *M. leprae* (Fig. 2, middle panel) [8], [16]. However, simultaneous clofazimine treatment and *M. leprae* infection of THP-1 cells led to decreased ADRP and increased HSL mRNA levels (Fig. 2, right panel). The decrease of ADRP and increase of HSL mRNA expression were further confirmed by quantitative real-time PCR (Fig. S3), which also supports the linearity of our RT-PCR data. Thus, it was shown that clofazimine modulated expression of ADRP and HSL only in *M. leprae*-infected cells. Similar results were observed for ADRP and HSL protein expression levels in each experiment.

### Clofazimine antagonizes the effects of *M. leprae* to modulate ADRP and HSL expression

**Figure 3:**
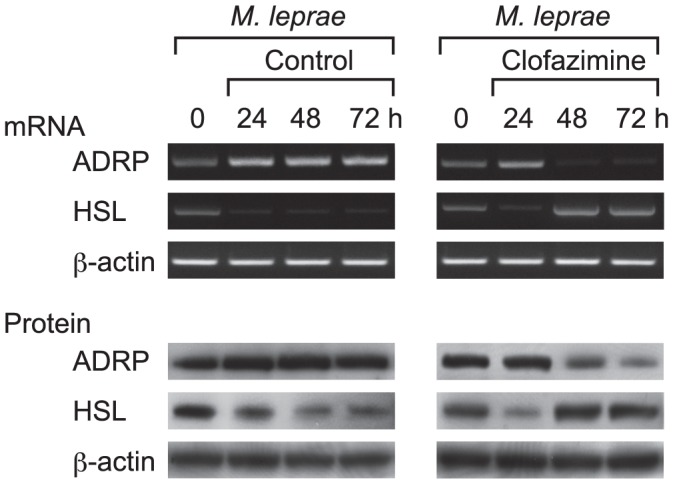
Clofazimine counteracts *M. leprae* to modulate ADRP and HSL expression levels. THP-1 cells were cultured in six-well plates and infected with *M. leprae* (MOI = 10) for 24 h. Clofazimine (2.0 µg/ml) was added and incubation continued another 24 and 48 h (48 and 72 h from *M. leprae* infection). Total RNA and total cellular protein were purified and RT-PCR and Western blot analyses of ADRP, HSL and β-actin were performed. Representative results from three independent experiments are shown.

In the above studies, THP-1 cells were simultaneously treated with clofazimine and infected with *M. leprae*. Therefore, there was a possibility that clofazimine might have modulated the cellular environments of THP-1 cells before engulfing *M. leprae*. To eliminate this possibility and to imitate clinical situations, THP-1 cells were first infected with *M. leprae* for 24 h, to allow cells to engulf enough bacilli, before they were treated with clofazimine. *M. leprae* infection enhanced ADRP expression and suppressed HSL expression for up to 72 h (Fig. 3, left panel), which is consistent with the results shown in Fig. 2, middle panel. However, adding clofazimine 24 h after *M. leprae* infection produced lower levels of ADRP expression, but increased HSL expression (Fig. 3, right panel). Interestingly, ADRP expression fell even lower than the original level, and HSL rose higher than original levels, following clofazimine treatment. These results suggest that the lipid catabolic activity once suppressed by *M. leprae* infection was reactivated by clofazimine treatment, which in turn would promote lipolysis in infected macrophages and decrease cellular lipids. Also, these results are consistent with clinical situations in which HSL mRNA levels were recovered following successful treatment with MDT in LL and BL patients [16].

### Clofazimine increases expression of IFN-β and IFN-γ mRNA in *M. leprae*-infected THP-1 cells

**Figure 4:**
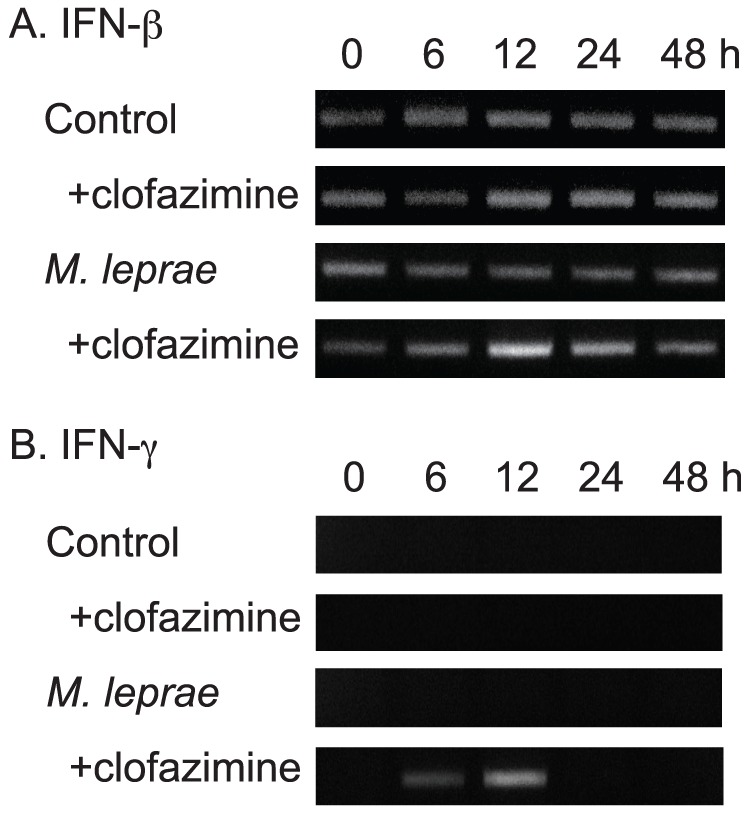
Clofazimine increases mRNA expression of IFN-β and IFN-γ in *M. leprae*-infected THP-1 cells. THP-1 cells were cultured in six-well plates with or without 2.0 µg/ml clofazimine in the presence or absence of *M. leprae* infection (MOI = 10). After incubating for the indicated period of time, total RNA was purified and RT-PCR analysis of IFN-β (A) and IFN-γ (B) was performed. Representative results from three independent experiments are shown.

The decrease in ADRP expression and increase in HSL expression produced by clofazimine treatment were also observed when *M. leprae*-infected cells were further treated with peptidoglycan (PGN), a ligand for Toll-like receptor (TLR)-2, to activate innate immunity [8], [16]. We therefore hypothesized that clofazimine treatment might activate the innate immune response of THP-1 cells, which also confers bactericidal activities. To assess activation of innate immunity, production of interferon IFN-β and IFN-γ mRNA was evaluated in control and *M. leprae*-infected THP-1 cells treated with clofazimine. A transient increase of IFN-β and induction of IFN-γ were observed only in THP-1 cells infected with *M. leprae* and treated with clofazimine (Figs. 4A and 4B). Transient induction of IFNs as a result of macrophage activation is consistent with previous reports [22]–[24]. Innate immune activation of infected cells will further contribute to the elimination of intracellular bacilli, which is also consistent with the observation that the active form of vitamin D suppresses CORO1A expression in THP-1 cells [21].

### Clofazimine treatment decreases the cellular lipid droplets in *M. leprae*-infected THP-1 cells

**Figure 5:**
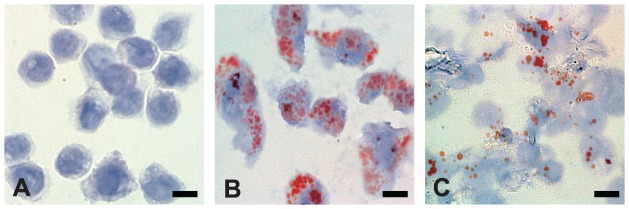
Clofazimine decreases cellular lipid accumulation in *M. leprae*-infected THP-1 cells. THP-1 cells were grown on glass coverslips in 24-well plates. Cells with no treatment (A), infected with *M. leprae* (MOI = 10) (B), and infected with *M. leprae* (MOI = 10) and treated with clofazimine (2.0 µg/ml) (C) were cultured for 48 h. Oil-red-O staining followed by brief hematoxylin counter staining was performed and observed under a microscope. Representative results from three independent experiments are shown. Bars = 10 µm.

To test whether the decrease in ADRP expression and increase in HSL expression after clofazimine treatment would result in less accumulation of cellular lipids after *M. leprae* infection, THP-1 cells were infected with *M. leprae* (MOI = 10) in the presence or absence of 2.0 µg/ml clofazimine for 48 h. Oil-red-O staining clearly demonstrated the accumulation of cellular lipid droplets following *M. leprae* infection (Fig. 5B vs. Fig. 5A). In *M. leprae*-infected cells treated with clofazimine, the amount of lipid droplets in the cell had significantly decreased by 48 h (Fig. 5C vs. 5B). The decrease in cellular lipid droplets is in agreement with the results shown in this study in which clofazimine decreased ADRP and increased HSL expression in *M. leprae*-infected cells.

### ADRP and HSL expression levels in skin lesions correlate with the clinical course of leprosy before and after treatment of leprosy patients

**Figure 6:**
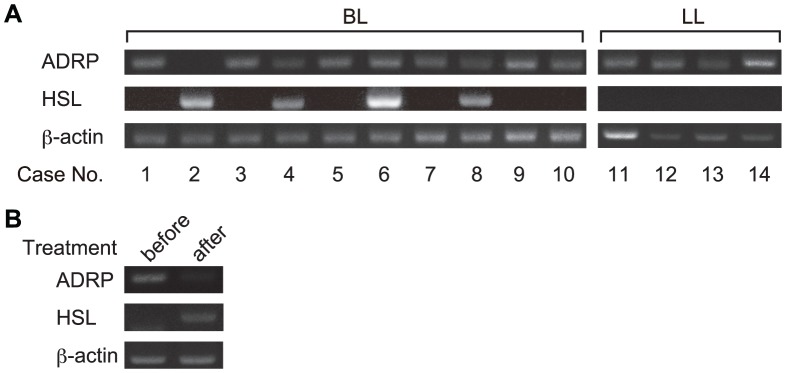
Detection of ADRP and HSL mRNA in slit-skin smear samples from leprosy patients. Total RNA was isolated from slit-skin smear specimens taken from ten BL and four LL patients (A) or from one patient before and after treatment (B). Total RNA was purified and RT-PCR analysis of ADRP, HSL and β-actin was performed. Representative results from three independent experiments are shown.

To confirm the expression pattern of ADRP and HSL in clinical courses of leprosy, ADRP and HSL mRNA levels were evaluated in slit-skin smear specimens by RT-PCR analysis. ADRP mRNA was detected in all LL and most BL cases tested (Fig. 6A, right panel). HSL mRNA was detected in four BL cases; however, ADRP mRNA expression in these cases was absent or weaker than in other BL samples (Fig. 6A, cases 2, 4, 6 and 8). In one case, from which serial samples were obtained, the expression of ADRP mRNA decreased and HSL mRNA levels increased after treatment (Fig. 6B).

**Figure 7:**
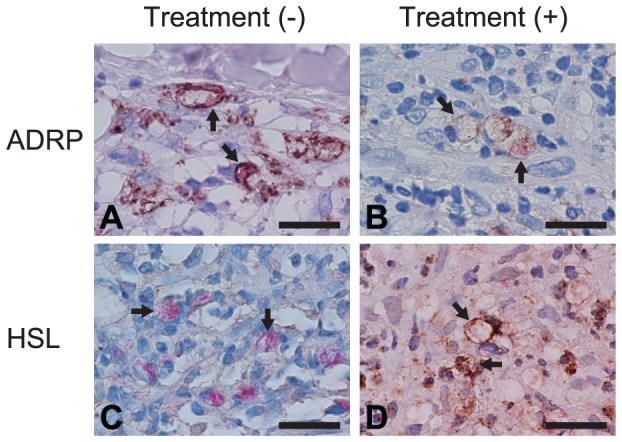
Immunostaining of ADRP and HSL proteins in skin biopsy specimens before and after treatment. Sections of skin biopsy specimens taken from one patient before (A and C) and after (B and D) treatment were subjected to immunostaining of ADRP (A and B) and HSL (C and D), followed by acid-fast staining for *M. leprae* and hematoxylin counterstaining. Arrows indicate phagosome membrane that contains *M. leprae*. Representative results from three independent experiments are shown. Bars = 20 µm.

To further confirm changes in ADRP and HSL expression following treatment, immunohistochemical and acid-fast staining were performed using formalin-fixed paraffin-embedded skin tissue sections. Consistent with a previous report, ADRP localized to phagosome membranes that contains solid-shaped *M. leprae* (Fig. 7A) [8]. HSL staining was not evident before treatment (Fig. 7C). Three months after treatment, staining of the bacilli showed a dotted pattern with no solid-staining, indicating degeneration of *M. leprae* (Fig. 7 B and 7D). At this point, ADRP staining was faint (Fig. 7B), but strong HSL staining was observed along the phagosomal membrane (Fig. 7D). These staining patterns correlate with changes in mRNA levels of ADRP and HSL in the skin smears (Fig. 6B).

## Discussion

In previous studies, we showed that *M. leprae* infection increases ADRP expression and decreases HSL expression in host macrophages [8], [16]. The results of the present study demonstrate that clofazimine, one of the three major drugs used to treat leprosy, counteracts the effect of *M. leprae* to reduce ADRP and increase HSL expression of both mRNA and protein levels. These results are consistent with our observations in clinical samples obtained from leprosy patients, in which HSL levels were not detectable in skin smear specimens before treatment, but re-appeared shortly after MDT [8], [16]. The other two MDT drugs, dapsone and rifampicin, revealed no effects on the expression of either ADRP or HSL.

Mycobacteria survive by evading the host immune system and accessing host metabolic pathways to obtain nutrients for growth. *M. leprae* has undergone reductive evolution and pseudogenes now occupy half of its genome [25]–[27], thus *M. leprae* is thought to be the mycobacterium most dependent on host metabolic pathways, including host-derived lipids. As we previously reported, PGN can activate TLR2 to increase the expression of HSL [16] and suppress ADRP and perilipin expression [7], [8], [21]. These effects mediated by the TLR-initiated signaling pathway will induce lipid degradation, which makes it difficult for *M. leprae* to survive within host cells. *M. leprae* infection not only suppresses HSL expression, but also invalidates all effects of PGN on ADRP and perilipin, thus ensuring a phagosome environment that is favorable for mycobacterial survival [16]. In the present study clofazimine increased HSL expression and decreased ADRP expression only in *M. leprae*-infected cells. The amounts of lipids accumulated in the cells decreased when clofazimine was added to the cell culture medium. The decrease of the lipid-rich environment against the survival of *M. leprae* may be one of the key actions of clofazimine.

Clofazimine was the first clinically developed riminophenazine for the treatment of tuberculosis [28]. Its use has been extended to many Gram-positive bacterial infections as well as mycobacterial diseases [28]–[30]. The drug is now widely used for the treatment of leprosy, but its mechanism remains unclear [31]–[33]. The drug is extremely lipophilic and is also active in membrane destabilization and possible promotion of antigen processing. Stimulated phospholipase A2 activity and subsequent accumulation of arachidonic acid and lysophospholipids were confirmed in clofazimine-induced membrane destabilization [29], [34]. Increased major histocompatibility complex (MHC) class II expression in peripheral blood monocytes [35], up-regulated lysosomal enzyme activity of cultured macrophages [36] and decreased suppressor T-cell activity in mycobacteria-infected mice [37] reveal the potential role of clofazimine in facilitating immune recognition.

Although the underlying molecular mechanisms are not clear, clofazimine suppressed ADRP and induced HSL, IFN-β and IFN-γ expression only in cells infected with *M. leprae*, the same effects products by PGN [8], [16], [21]. Therefore, it is possible that clofazimine revives at least some of the activities of PGN, which is normally shielded by redundant mycolic acid at the *M. leprae* cell wall. Given the extreme lipophilicity of clofazimine and its activity against many Gram-positive bacteria, clofazimine may interact with the mycolic acid in the *M. leprae* cell wall that facilitates the exposure of PGN, which in turn activates TLR2-mediated signaling cascades, subsequently decreasing ADRP and increasing HSL [8], [16], [21]. Furthermore, since most lepra reactions, a cell-mediated, delayed-type hypersensitivity immune response, occur during or after MDT [38], [39], the prospect that clofazimine rescues shielded PGN activities, promoting lysosomal fusion and antigen processing, would be a plausible explanation for the trigger of lepra reactions.

The results from present and previous studies may explain the underlying mechanisms, at least in part, of successful parasitization of *M. leprae* and the effects of MDT treatment observed in patients. In conclusion, we have shown that clofazimine devastates the lipid-rich environment in *M. leprae*-infected host macrophages by modulating the expression of ADRP and HSL and activates the innate immune response of infected cells, both of which would be important in fighting mycobacterial infection.

## Supporting Information

Figure S1 **Quality of RNA samples purified from THP-1 cells infected with** ***M. leprae*** **.** RNA samples were purified from THP-1 cells infected with *M. leprae* as described in the Materials and Methods. Ten samples were analyzed using a 1% denatured agarose gel (A) and four were analyzed with theAgilent 2100 Bioanalyzer (Agilent Technologies, Santa Clara, CA) (B).
(EPS)

Figure S2 **Linearity of RT-PCR analysis.** RNA samples were serially diluted and RT-PCR analysis of ADRP, HSL and β-actin was performed. Specific bands on the agarose gel were quantified using ImageJ64 software.
(EPS)

Figure S3 **The effect of simultaneous clofazimine treatment and** ***M. leprae*** **infection on mRNA levels in THP-1 cells.** THP-1 cells were cultured in six-well plates with or without 2.0 µg/ml clofazimine in the presence of *M. leprae* infection (MOI = 10). After incubating for the indicated period of time, total RNA was purified and real-time PCR analyses of ADRP (A), HSL (B) and β-actin were performed as previously described (reference 8). The same primers that were used for RT-PCR analysis were utilized with SYBER Green PCR Master Mix (Applied Biosytems). All samples were amplified in triplicate from the same RNA preparation. Each result is expressed as the mean ± SE. The Student's t-test was used for statistical analysis. One asterisk indicates a value of P<0.05; two asterisks indicate a value of P<0.01; and three asterisks indicate a value of P<0.001.
(EPS)
